# Multimodal and Multiscale Characterization of the Bone‐Bacteria Interface in a Case of Medication‐Related Osteonecrosis of the Jaw

**DOI:** 10.1002/jbm4.10693

**Published:** 2022-11-08

**Authors:** Chiara Micheletti, Liza‐Anastasia DiCecco, Cecilia Larsson Wexell, Dakota M. Binkley, Anders Palmquist, Kathryn Grandfield, Furqan A. Shah

**Affiliations:** ^1^ Department of Materials Science and Engineering McMaster University Hamilton Ontario Canada; ^2^ Department of Biomaterials, Sahlgrenska Academy University of Gothenburg Gothenburg Sweden; ^3^ Department of Oral and Maxillofacial Surgery Skåne University Hospital Lund Sweden; ^4^ Department of Oral and Maxillofacial Surgery and Oral Medicine Malmö University Malmö Sweden; ^5^ School of Biomedical Engineering McMaster University Hamilton Ontario Canada

## Abstract

Medication‐related osteonecrosis of the jaw (MRONJ) is a known side effect of bisphosphonates (BPs). Although bacterial infection is usually present, the etiology of MRONJ remains unknown. Here we apply a multimodal and multiscale (micro‐to‐nano) characterization approach to investigate the interface between necrotic bone and bacteria in MRONJ. A non‐necrotic bone sample was used as control. Both necrotic and non‐necrotic bone samples were collected from the jaw of a female individual affected by MRONJ after using BPs for 23 years. For the first time, resin cast etching was used to expose bacteria at the necrotic site. The bone–bacteria interface was also resolved at the nanoscale by scanning transmission electron microscopy (STEM). Nanosized particulates, likely corresponding to degraded bone mineral, were often noted in close proximity to or enclosed by the bacteria. STEM also revealed that the bone–bacteria interface is composed of a hypermineralized front fading into a highly disordered region, with decreasing content of calcium and phosphorus, as assessed by electron energy loss spectroscopy (EELS). This, combined with the variation in calcium, phosphorus, and carbon across the necrotic bone–bacteria interface evaluated by scanning electron microscopy (SEM)‐energy dispersive X‐ray spectroscopy (EDX) and the lower mineral‐to‐matrix ratio measured by micro‐Raman spectroscopy in necrotic bone, indicates the absence of a mineralization front in MRONJ. It appears that the bone–bacteria interface originates not only from uncontrolled mineralization but also from the direct action of bacteria degrading the bone matrix. © 2022 The Authors. *JBMR Plus* published by Wiley Periodicals LLC on behalf of American Society for Bone and Mineral Research.

## Graphical abstract

Multimodal and multiscale characterization of the bone–bacteria interface in a case of medication‐related osteonecrosis of the jaw is presented. Bacteria in the biofilm were imaged by secondary electron scanning electron microscopy (SE‐SEM) after resin cast etching. The bone–bacteria interface was resolved at the nanoscale using high‐angle annular dark‐field scanning transmission electron microscopy (HAADF‐STEM). The schematic was created using icons from BioRender.com.

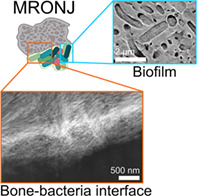

## Introduction

Skeletal disorders such as osteoporosis and cancer‐related bone metastasis are often treated with bisphosphonates (BPs) because of their antiresorptive function.^(^ ^1^ ^)^ By inhibiting osteoclastic activity and by binding to hydroxyapatite crystals, BPs inhibit hydroxyapatite breakdown^(^ ^2^ ^)^ and alter the balance in bone remodeling by suppressing bone resorption^(^ ^3^ ^)^ in favor of a net gain in bone mineral density (BMD).^(^ ^4^ ^)^ However, treatment with BPs for a long period of time or in a high dosage has been associated with a greater risk of developing osteonecrosis of the jaw (ONJ),^(^ ^5^, ^6^, ^7^, ^8^, ^9^ ^)^ often referred to as medication‐related ONJ (MRONJ). The American Association of Oral and Maxillofacial Surgeons (AAOMS) classifies MRONJ as the presence in the jaw of exposed bone or bone that can be probed through an intra‐ or extraoral fistula that does not heal within 8 weeks after identification and appropriate intervention, in the absence of metastasis or history of radiation therapy to the craniofacial region.^(^ ^7^, ^10^, ^11^ ^)^ Histological examinations of individuals diagnosed with MRONJ confirm the presence of necrotic bone and often show signs of infection.^(^ ^12^ ^)^ The pathogenesis of MRONJ remains unclear, but preclinical and clinical studies indicate that infection plays an important role, although it is still debated whether MRONJ precedes or follows oral infection.^(^ ^13^, ^14^, ^15^ ^)^

Overall, it appears that several factors contribute to the onset of MRONJ. Infection in the oral cavity can be triggered by dental traumas and procedures, for example, tooth extraction, which are often needed to resolve the damage caused by previous infections (e.g., dental caries) or diseases such as periodontitis.^(^ ^16^ ^)^ Infection and, consequently, microbial biofilm formation can lead to bone destruction despite the presence of BPs.^(^ ^15^ ^)^ On the other hand, suppression of bone resorption by BPs may cause a lower bone turnover and a less responsive (“frozen”) bone surface incapable of adequately contrasting microbial colonization.^(^ ^6^, ^15^ ^)^ High local concentrations of BPs can be toxic to the soft tissue and to cells involved in the local immune response, in turn compromising the healing of the oral mucosa and leading to persistent infection.^(^ ^15^, ^17^ ^)^ Wound healing could even be worsened by compromised angiogenesis, which is sometimes reported for BPs.^(^ ^13^ ^)^ It is unclear whether microbial biofilm pathogens are able to directly resorb bone, but some in vitro and ex vivo work has presented such a possibility.^(^ ^18^ ^)^ In other diseases of the oral cavity such as periodontitis, microbial biofilms play a critical role in tissue breakdown.^(^ ^16^, ^19^ ^)^ The likelihood of developing MRONJ under BP treatment probably depends on a combination of environmental, metabolic, and genetic factors, with impaired general condition, systemic chronic inflammation, diabetes, smoking, and obesity often seen as risk factors.^(^ ^11^, ^20^, ^21^ ^)^

Here we present a case study of a female individual diagnosed with MRONJ after a 23‐year‐long treatment with BPs (alendronate) for osteoporosis. Specifically, we focus on examining the interface between necrotic bone and bacteria from a structural and compositional point of view at different length scales, from the micro‐ to the nanoscale. As appropriate, necrotic bone was also compared to non‐necrotic bone retrieved from unexposed areas of the jaw. The materials science approach of multimodal, multiscale analytic characterization applied to a clinically relevant issue presented the opportunity to probe the relationship between MRONJ, bacterial infection, and bone mineralization from a less conventional perspective. This in turn offered new insights into possible events taking place where necrotic bone and bacteria meet, including a potentially direct role of bacteria in degrading the bone matrix, never demonstrated in vivo to date.

## Materials and methods

### Patient history

**Fig. 1:**
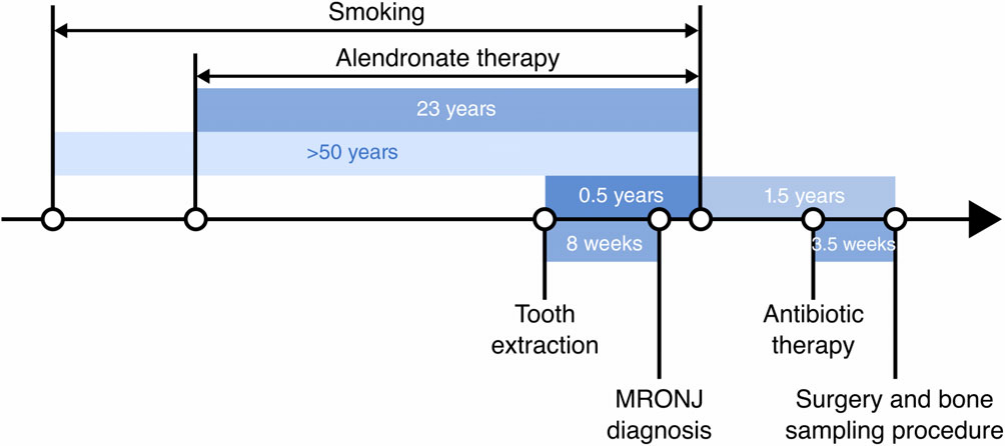
Timeline (not to scale) of clinically relevant events preceding bone sampling procedure due to medication‐related osteonecrosis of the jaw (MRONJ) diagnosis.

Necrotic and non‐necrotic bone samples were collected from the jaw of a 73‐year‐old osteoporotic female with a low body mass index (BMI) (<18 kg/m^2^) and 23 years of exposure to BPs (alendronate tablets) administered orally, suffering from MRONJ in both the upper and lower jaws. A schematic timeline of clinically relevant events is presented in Figure 1. Two years before the bone sampling procedures, the upper right second premolar and the upper right canine were extracted (Figure S1). Healing of the area was absent, and the patient was diagnosed with MRONJ at the upper right jaw. MRONJ was also present at both sides of the lower jaw. The patient stopped smoking (after over 50 years) and taking alendronate 1.5 years before the bone sampling procedure.

### Sample collection, histology, and microbiology

The patient was treated with 1.6 g phenoxymethylpenicillin orally for 3.5 weeks prior to surgery and sampling. Under general anesthesia, bone samples were collected from the exposed and necrotic areas (upper jaw and lower jaw, right side) (*n* = 2) and from intact/unaffected areas (upper jaw, left side) (*n* = 1) (Figure S1). In the upper jaw, a crestal incision was made from the left side close to the midline to the second upper molar on the right side. A large mobile sequester of bone, 2.5 × 2.5 × 2 cm^3^, was removed. Swabbing for microbiology analyses was made directly at the bottom of the lesion. A small cortical bone sample was collected from the non‐exposed area on the left side of the upper jaw. In the lower jaw, six teeth were extracted. After a crestal incision and uncovering of the jawbone, large areas of necrotic tissue were noted and removed.

The samples were fixed by immersion in 10% neutral buffered formalin, dehydrated, and resin embedded (LR White, London Resin Company, UK). Toluidine blue–stained ground sections were prepared by sawing and grinding to a thickness of 30–40 μm for histological examination in a light microscope (Nikon Eclipse E600) using a condenser with numerical aperture (NA) set to 1 and ×4, ×10, ×20, and ×40 objectives (0.13, 0.3, 0.5, and 0.75 NA, respectively).

The study was approved by the Regional Ethical Review Board of Gothenburg (Dnr 424–08), including sampling from apparently non‐necrotic areas, as part of the intervention plan for dental restoration established by the clinician.

### Scanning electron microscopy and energy dispersive X‐ray spectroscopy

Backscattered electron (BSE) scanning electron microscopy (SEM) images were acquired in an environmental SEM (Quanta 200, FEI Company, The Netherlands) operated at 20 kV accelerating voltage, 1 Torr water vapor pressure, and 10 mm working distance. The same instrument settings were used for energy dispersive X‐ray spectroscopy (EDX). Three line scans across the bone–resin interface were acquired for each sample using 20 accumulations per spectrum (AZtec 5.0, Oxford Instruments, UK). The interaction area and volume for the elements considered (C, Ca, and P) were estimated to range between 53–66 μm^2^ and 183–257 μm^3^, respectively. These values were obtained from the probed radius and volume for non‐backscattered electrons (here considered as the range upper limit, as an approximation for C), and P and Ca (here considered as the range lower limit) computed by Howell and Boyde.^(^ ^22^ ^)^ The interaction area was estimated as the area of a circle using the radius computed by Howell and Boyde.^(^ ^22^ ^)^ EDX data were smoothed by Savitzky–Golay filtering using the “savgol_filter” function (21 coefficients, fourth‐order polynomial) available in the “scipy.signal” library in Python 3.8.10. The width of the region where C, Ca, and P presented the highest variation was measured as the width of the peak in the first‐order derivative of the unsmoothed data, computed using the “savgol_filter” function (21 coefficients, fourth‐order polynomial, first‐order derivative order). The region of the C, Ca, and P plots where significant variation occurred was fitted with a line using “numpy.polyfit” in Python 3.8.10 in order to obtain the slope of the line.

### Micro‐Raman spectroscopy

Micro‐Raman spectroscopy was performed using a confocal Raman microscope (Renishaw inVia Qontor) equipped with a 633 nm laser and LiveTrack focus‐tracking technology. The laser was focused on the surface using a ×100 objective (0.9 NA). Following the definitions of lateral resolution^(^ ^23^ ^)^ $\left(∆x=0.61\lambda /\mathrm{NA}\right)$ and depth of focus^(^ ^23^, ^24^ ^)^ $\left(∆z=\pm \frac{4.4\mathrm{n\lambda}}{2\pi {\left(\mathrm{NA}\right)}^{2}}\right)$, the lateral (Δx) and vertical (2|Δz|) resolutions were determined to be equal to 0.4 and 1.1 μm, respectively, resulting in a probed volume (half‐ellipsoid) of 0.7 μm^3^. Line scans of 11 points each were acquired at 1 μm step size across the bone–resin interface. Each point measurement was acquired with a 3 s exposure time and five accumulations per spectrum and preceded by a 60 s bleaching. Five line scans per sample were acquired in different locations. Spectra were denoised by fast Fourier transform (FFT) using the “scipy.fft” module available in the “scipy” library in Python 3.8.10. The noise threshold was set as the average signal in the 340–400 cm^−1^ region of the spectrum multiplied by a constant value *k* (*k* = 2.5 in non‐necrotic bone sample; *k* = 5 in necrotic bone samples). Spectra were then truncated (700–1050 cm^−1^) and baseline‐subtracted by fitting a fifth‐order polynomial using the “baseline_poly” function available in the “rampy” library in Python 3.8.10. Mineral content in the collagen matrix was estimated as the mineral‐to‐matrix ratio, taken as the ratio between the peak intensity in the phosphate band (*ν*_1_ PO_4_^3*−*^, 930–980 cm^−1^) and in the phenylalanine band (Phe, 1000–1005 cm^−1^).^(^ ^25^ ^)^ Mineral‐to‐matrix ratios computed from spectra acquired in the bone region (*n* = 5) were averaged in each line scan and then averaged for each sample.

### Resin cast etching

Samples were immersed in 9% H_3_PO_4_ for 30 s, quickly rinsed in deionized H_2_O (for 2 s), immersed in 5% NaOCl for 5 min, rinsed again in deionized H_2_O for 30 s, and allowed to air‐dry overnight. After Au sputter coating (~10 nm thickness), secondary electron (SE) imaging was carried out in a high‐vacuum SEM (Ultra 55 FEG, Leo Electron Microscopy, UK) operated at 3 kV.

### Nano‐analytical electron microscopy

An electron transparent sample of necrotic bone interfacing with the bacteria‐invaded resin for transmission electron microscopy (TEM) and scanning TEM (STEM) analyses was prepared by in situ lift‐out in a dual‐beam focused ion beam (FIB) instrument (Zeiss NVision 40, Carl Zeiss AG, Oberkochen, Germany) equipped with a 30 kV Ga ion column, a gas injection system, and a micromanipulator (Kleindiek Nanotechnik GmbH, Reutlingen, Germany), following published protocols.^(^ ^26^ ^)^ The region of interest for the in situ lift‐out was selected by prior BSE‐SEM imaging in a high‐vacuum SEM (FEI Magellan 400 XHR, Thermo Fisher Scientific, Hillsboro, OR, USA) operated at 10 kV, after the samples were sputter coated with Au (~10 nm thickness).

TEM and STEM imaging were performed at 200 kV (Talos 200X, Thermo Fisher Scientific, Waltham, MA, USA) and 300 kV (FEI Titan 80–300 LB, Thermo Fisher Scientific, Hillsboro, OR, USA), respectively. TEM images were collected in bright field (BF) mode. STEM images were acquired using a high‐angle annular dark‐field (HAADF) detector.

#### Particulate analysis

Particles observed in HAADF‐STEM images in the bacteria‐invaded region were measured using Fiji^(^ ^27^ ^)^ (NIH, Bethesda, MD, USA) with the “Analyze particles” command after applying a bandpass filter and a threshold. The mean diameter of each particle was obtained by averaging the major and minor axes of the ellipse fitted to the particle by the software. The average particle size was computed by averaging the mean diameter of 127 distinct particles.

#### Collagen banding measurement

The average D‐spacing in the collagen banding pattern was measured from HAADF‐STEM images using Fiji^(^ ^27^ ^)^ (NIH, Bethesda, MD, USA). Five fibrils in different locations were examined. For each fibril, the histogram profile was extracted from a 20‐pixel‐thick line (0.85–1.19 nm pixel size). The average length of the period (i.e., overlap and gap regions) in each fibril was obtained by dividing the total length between the first and sixth minima in the histogram profile by the number of periods included, that is, five. The average D‐spacing was computed as the mean of the values measured for each fibril.

#### Electron energy loss spectroscopy

Electron energy loss spectroscopy (EELS) was performed in a STEM instrument (FEI Titan 80–300, Thermo Fisher Scientific, Hillsboro, OR, USA) equipped with a Gatan Tridiem spectrometer (Gatan, Inc., Pleasanton, CA, USA). The microscope was operated at 300 kV with an 8 mrad convergence semi‐angle and a 25 mrad collection semi‐angle. EELS maps across the bone–bacteria interface were acquired with an energy dispersion of 0.5 eV/channel, a 0.05 s exposure time, and a 5 nm step size. EELS spectral images were denoised by principal component analysis (PCA) and background‐subtracted in Gatan Microscopy Suite (GMS) 3.4.3 (Gatan, Inc., Pleasanton, CA, USA). Elemental maps for C, Ca, and P were obtained by extracting the signal in the peak energy window of each element. From the elemental maps, variation in elemental concentration across the interface was obtained by considering a 10‐pixel‐thick line scan.

#### Selected area electron diffraction

Selected area electron diffraction (SAED) patterns were acquired using a 40 μm aperture (illuminating an area of about 250 nm in the sample) in a TEM instrument (Talos 200X, Thermo Fisher Scientific, Waltham, MA, USA) operated at 200 kV.

### Statistical analysis

Final values of average measurements are reported as mean values ± standard deviations. When comparing non‐necrotic and necrotic bone, statistical significance was evaluated by a Mann–Whitney U test (*α* = 0.05) in Python 3.8.10 using the “scipy.stats” library.

## Results and Discussion

### Clinical and microscale manifestation of MRONJ

**Fig. 2:**
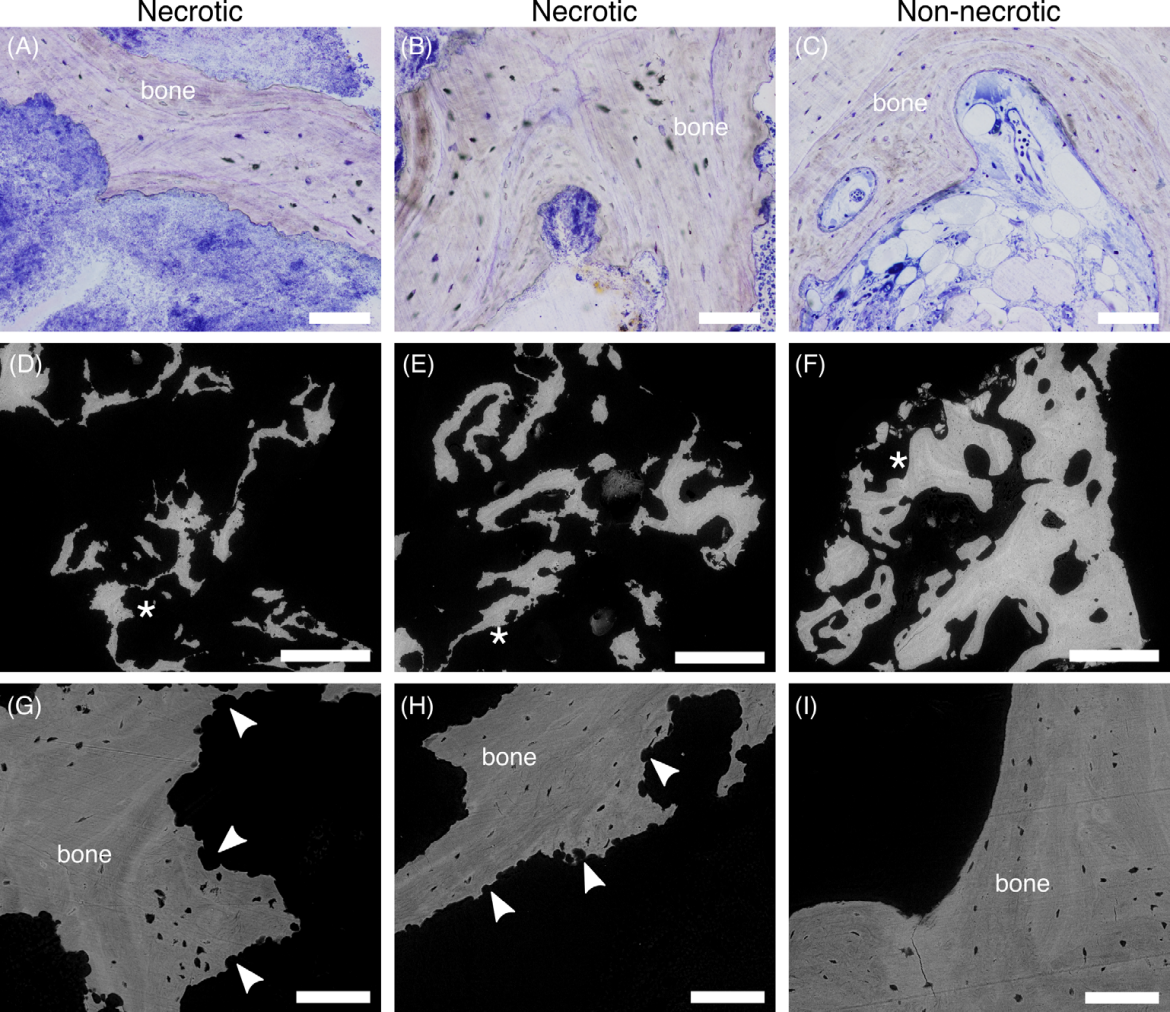
Histology and BSE‐SEM. Histological sections of necrotic (A, B) and non‐necrotic (C) bone (images acquired with a ×20 objective). The space interfacing with bone is occupied by an extensive biofilm in necrotic bone, whereas bone marrow surrounds non‐necrotic bone. Empty osteocyte lacunae in necrotic bone are indicated by absence of staining. Overview BSE‐SEM images of necrotic (D, E) and non‐necrotic (F) bone, and higher magnification images (G and H for necrotic bone, I for non‐necrotic bone), corresponding to areas indicated by asterisk in images D, E, and F. Necrotic bone displays an irregular surface (G and H, arrowheads), as opposed to the smooth contour of non‐necrotic bone (I). Images A, D, and G correspond to necrotic bone in the lower jaw, whereas images B, E, and H correspond to necrotic bone in the upper jaw. Scale bars are 100 μm in A, B, C, G, H, and I, and 1 mm in D, E, and F.

Histology confirmed the necrosis of the affected areas in the jaw, with most vascular spaces filled with bacteria rather than blood cells, and empty osteocyte lacunae, which is a characteristic histological trait of MRONJ^(^ ^28^ ^)^ (Figure 2A, B, Figure S2). On the other hand, bone from the unaffected area appears normal, that is, non‐necrotic (Figure 2C, Figure S2). Microbial swab demonstrated the abundant growth of *Neisseria* and *Prevotella*, including black pigmented fusiform rods, and the relatively abundant growth of *Haemophilus*‐like rods.

As was already visible in histological images, the bone surface resolved in BSE‐SEM presented a jagged outline in bone retrieved from necrotic areas (Figures 2D, E, G, H, Figure S3), whereas the contour was generally smooth in non‐necrotic bone (Figures 2F, I, Figure S3). The presence of irregular, scalloped borders has often been reported for MRONJ.^(^ ^12^, ^14^ ^)^ However, this surface appearance remains poorly understood and rather paradoxical because such irregular edges are usually indicative of ongoing osteoclast‐mediated resorption, which should be largely suppressed by BPs.^(^ ^3^ ^)^ Moreover, if the irregular edges were due to resorption only, this would not explain the difference between the surface appearance of necrotic and non‐necrotic bone. Therefore, we propose that it can be explained by an active role of bacteria in the degradation of bone extracellular matrix, as discussed subsequently.

Closer examination by BSE‐SEM of some osteocyte lacunae in necrotic bone revealed the presence of mineral inclusions, most likely hydroxyapatite‐based. Some rhomboidal nodules, analogous in shape and size to magnesium whitlockite crystals previously reported,^(^ ^29^ ^)^ were also noted (Figure S4). Although magnesium whitlockite has been described for pathological calcification in various tissues,^(^ ^30^ ^)^ whether BPs (in)directly contribute to mineralization in whitlockite rather than apatite remains to be determined.

**Fig. 3:**
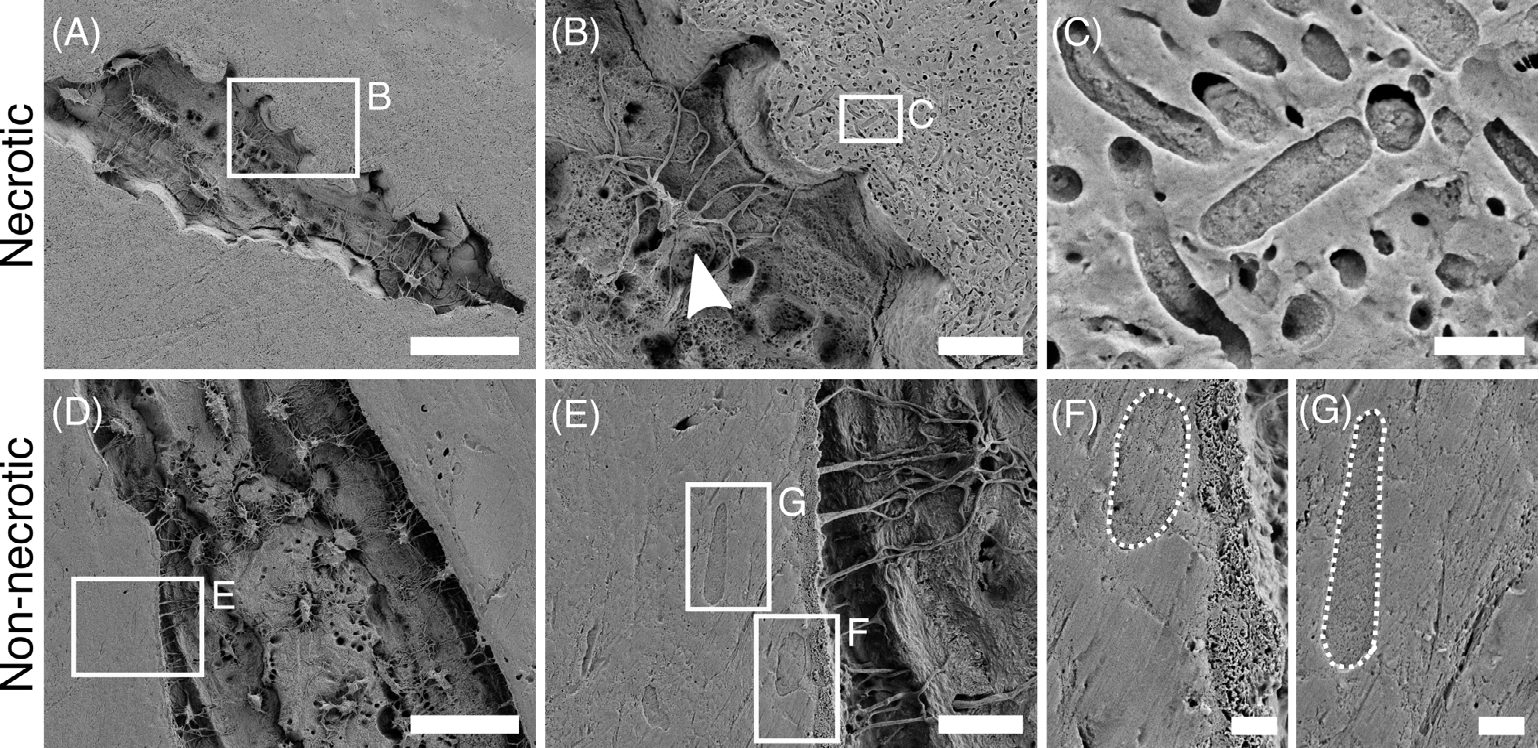
Resin cast etching. Overview SE‐SEM image of necrotic bone after resin cast etching (A), surrounded by an extensive biofilm (B) where several rod‐shaped bacteria can be distinguished (C). An osteocyte with “withered” protrusions, which could be indicative of a non‐vital cell, can also be observed (arrowhead in B). Overview SE‐SEM image of non‐necrotic bone after resin cast etching (D). At the bone surface, an osteoblast and a lining cell can be distinguished (E), marked by dotted line in higher magnification views in F and G, respectively. Scale bars are 50 μm in A and D, 10 μm in B and E, 1 μm in C, and 2 μm in F and G.

From SE‐SEM imaging after resin cast etching, bacteria were clearly visible in the resin region surrounding necrotic bone (Figure 3, Figure S5), corroborating the existence of an extensive biofilm^(^ ^31^ ^)^ (Figure 3A, B). No evidence of mineralized or mineral‐filled bacteria was found. Cocci, bacilli, and filamentous bacteria were observed (Figure 3C), in good agreement with the identification from the microbial swab and compatibly with rod‐shaped bacteria commonly present in MRONJ.^(^ ^14^, ^32^ ^)^ On the other hand, no bacteria were detected in the resin region in non‐necrotic bone (Figure 3D, E), but some osteoblasts (Figure 3F) and lining cells (Figure 3G) were identified instead in proximity to the bone surface, supporting the presence of bone deposition. Neither osteoblasts nor lining cells were spotted in the resin region interfacing with necrotic bone. However, this is not unexpected, since dead tissue is metabolically inactive. Some cells in necrotic bone appeared shrunken and with “withered” retracted protrusions, which could be indicative of non‐vital cells^(^ ^33^ ^)^ (Figure 3B). Little is known about osteocyte death in MRONJ,^(^ ^34^ ^)^ but it is believed that high intracellular concentrations of BPs can be toxic to bone cells other than osteoclasts.^(^ ^8^, ^13^ ^)^

### Ultrastructural considerations

**Fig. 4:**
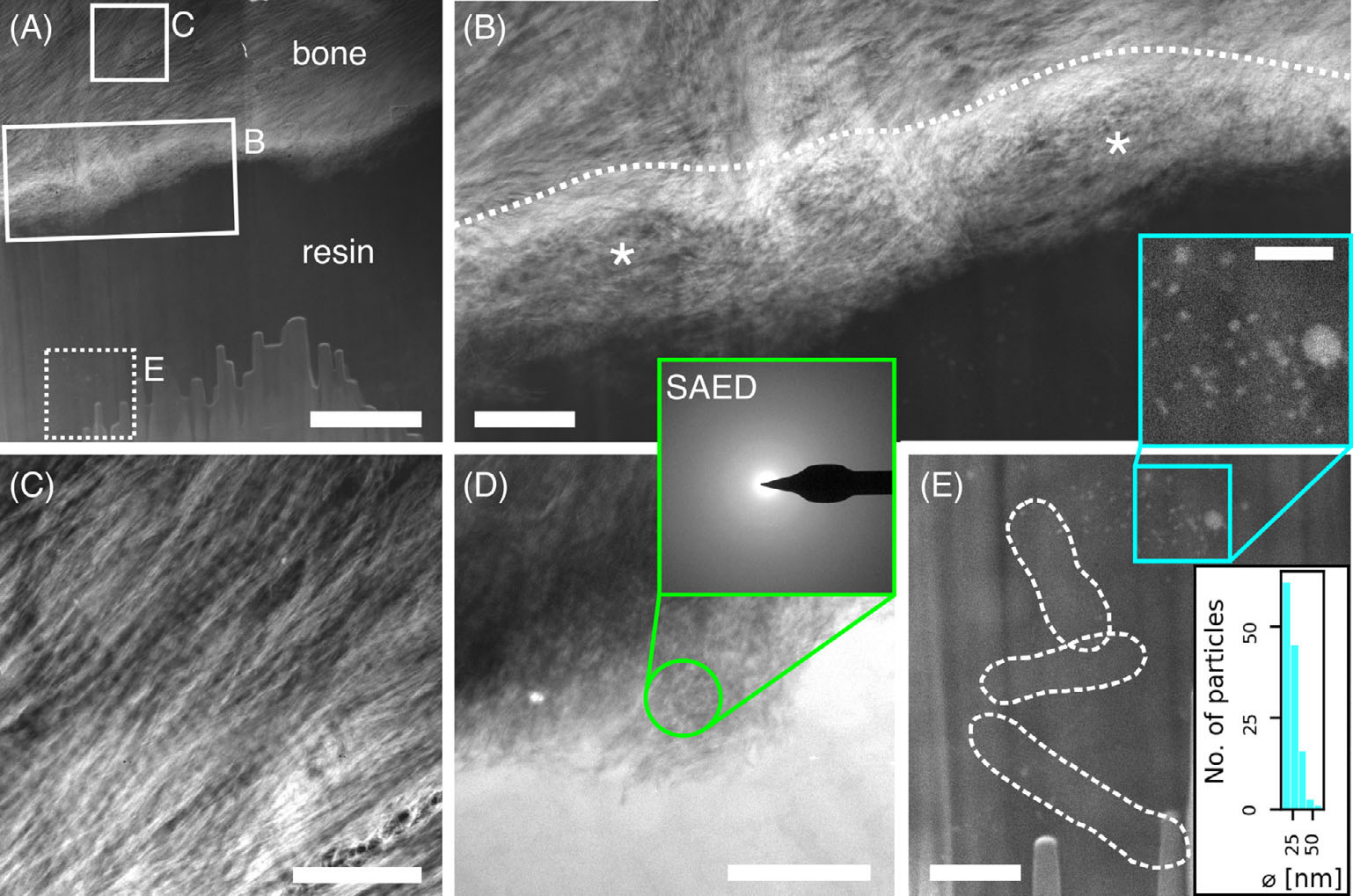
HAADF‐STEM and SAED. Overview HAADF‐STEM image of necrotic bone interfacing with bacteria‐invaded space (A). The interface appears as an electron‐dense band (dotted line) that fades into a loosely organized region (asterisk) toward the resin space (B, corresponding to area marked by rectangle in A). Further from the interface, bone structure appears normal, and the characteristic banding pattern of in‐plane collagen can be distinguished (C, corresponding to area marked by square in A). BF‐TEM image of disordered region at the interface (D), where bone appears lacking crystallinity, as indicated by absence of diffraction rings in the SAED pattern (inset with a green border, SAED from region marked by green circle in D). In the resin space (E, corresponding to area marked by dotted square in A), bacteria (dotted line, unmarked image available as Figure S7) and nanosized particulate matter (inset with a blue border) can be noted. The histogram in the bottom right corner of E represents the distribution of the particle diameter (⌀). Scale bars are 2 μm in A, 500 nm in B, C, D, and E, and 200 nm in the blue inset of E.

At the necrotic bone–resin interface, HAADF‐STEM images revealed the existence of a hypermineralized front, which fades into a region with exaggerated disorder and loosely arranged mineral platelets, and no discernable collagen fibrils, toward the resin space (Figure 4A, B). The hypermineralized front appears as an electron‐dense, high‐*Z*‐contrast band, approximately 100–350 nm in width, without any obvious structural features. Beyond this front, bone tissue displayed a normal structural organization, with in‐plane collagen fibrils originating a cross‐striated banding pattern with a 68 nm ± 1 nm D‐spacing (Figure 4C), compatibly to values commonly reported in the literature for healthy bone (i.e., 67 nm).^(^ ^35^, ^36^ ^)^ In the 400–500 nm‐wide disordered layer interfacing with the resin region, the complete absence of diffraction rings in SAED patterns suggests that this area is highly amorphous (Figure 4D), as opposed to that with in‐plane collagen fibrils exhibiting the characteristic (002) arcs of the *c*‐axis of hydroxyapatite (Figure S6).

In the resin region further away from bone, bacteria were observed (Figure 4E, Figure S7), confirming histological findings and SE‐SEM images of necrotic bone after resin cast etching. In addition, some high‐*Z*‐contrast particulate matter, 24 ± 8 nm in size, was noted often enclosed within or in close association with the bacteria (Figure 4E). We hypothesize that these nanosized particles are remainders of bone mineral recently having been degraded by the bacteria or by phagocytosing cells and, eventually, re‐expelled into the outer environment.

### Mineralization at the bone–bacteria interface

**Fig. 5:**
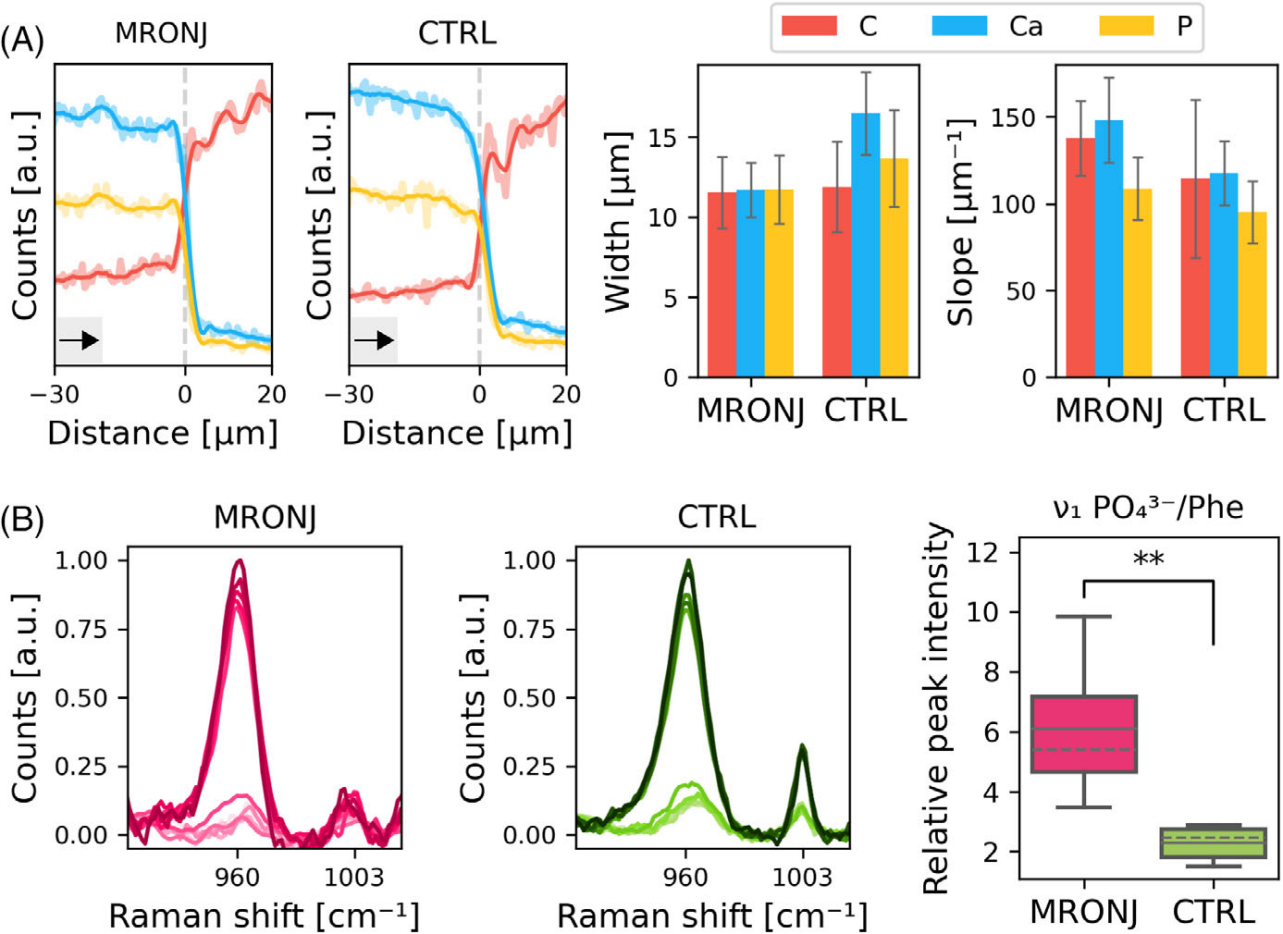
EDX and micro‐Raman spectroscopy. (A) EDX line scans of necrotic (MRONJ) and non‐necrotic (CTRL) bone taken across the bone–resin interface and bar graphs comparing the width and slope of the interface for the two groups. The difference was not statistically significant for either. The faded and bold lines in the line scan plots correspond to spectra before and after smoothing, respectively. The vertical dashed line in gray in the line scan plots approximately marks the interface between bone and resin. Black arrows indicate scanning direction, that is, from bone toward resin. (B) Raman spectra of points in line scans across the bone–resin interface for necrotic (MRONJ) and non‐necrotic (CTRL) bone and box plots showing mineral‐to‐matrix ratio (*ν*_1_ PO_4_^3*−*^/Phe) of the two groups (for points in bone region only). Boxes represent 50% of data, limited by upper and lower quartiles, and vertical lines indicate data range. The mean and median values are marked by continuous and dashed lines, respectively, across each box. ** denotes statistical significance (*p* < 0.01).

Trends in C, Ca, and P evaluated from SEM–EDX line scans revealed that all elements, and especially Ca, varied across a narrower region and at a faster rate (i.e., steeper slope of the fitted line) for the bone–resin interface in necrotic bone, although the difference with non‐necrotic bone was not statistically significant (*p* > 0.05) (Figure 5A, Table S1). Nonetheless, this appears to be in agreement with the fact that, although a mineralization front exists in non‐necrotic bone, and hence a gradual concentration gradient is expected, no such front is present in necrotic bone, which explains the higher abruptness in concentration change.

The mineral‐to‐matrix ratio was higher in necrotic than in non‐necrotic bone (*p* < 0.01), as evaluated from Raman spectra (Figure 5B, Table S2). Again, this can be explained by the lack of a mineralization front in the presence of necrosis, with no newly formed bone or osteoid‐rich regions that would contribute to the Phe signal, thereby lowering the mineral‐to‐matrix ratio. In addition, BP treatment has been associated with an increase in bone mineralization.^(^ ^4^ ^)^ From HAADF‐STEM images of necrotic bone, it is interesting to note that the collagen banding pattern was no longer distinguishable, whereas mineral was still present (albeit in a highly disordered state) at the bone–resin interface. The apparent absence of collagen (and, therefore, most of the organic component of the extracellular matrix) offers an explanation for the higher mineral‐to‐matrix ratio of necrotic bone.

**Fig. 6:**
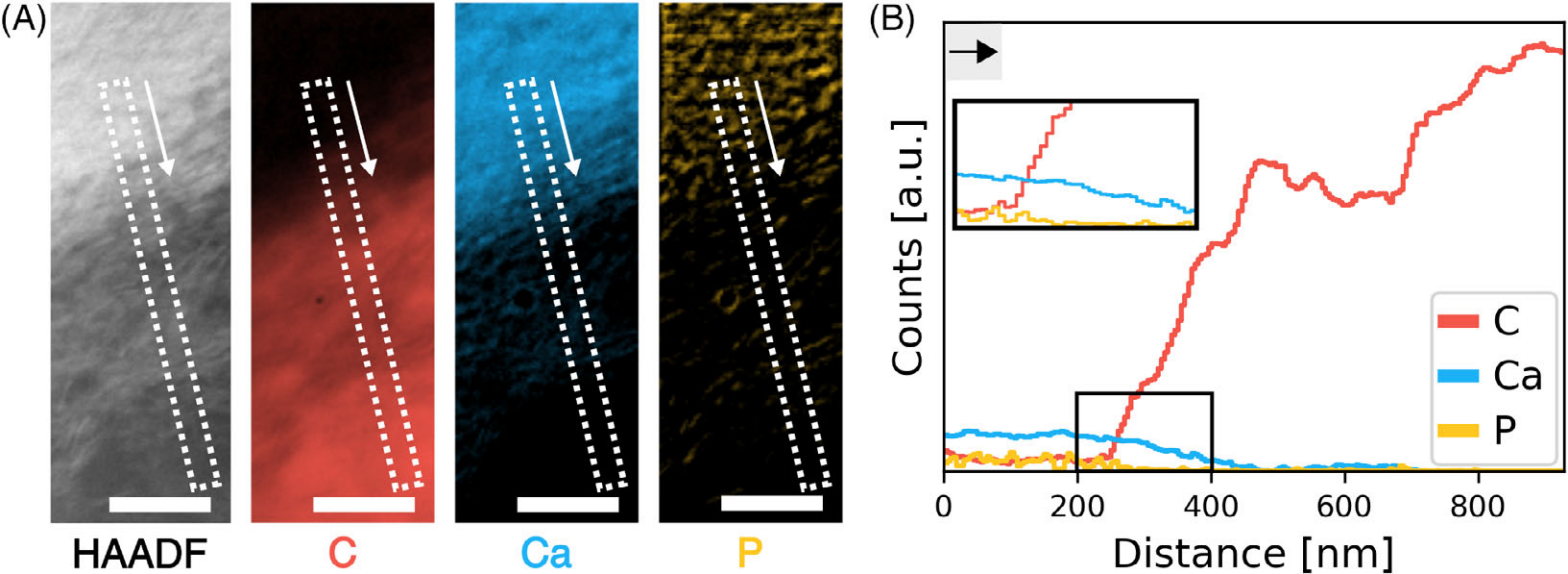
EELS. (A) HAADF‐STEM image and corresponding EELS maps for C, Ca, and P. (B) Variation in C, Ca, and P across the bone–resin interface extracted from the area marked by a dashed rectangle in the HAADF‐STEM image and EELS maps in A (white arrow indicates scanning direction, that is, from bone toward resin). The inset in B provides a magnified view of decrease in Ca and P across the interface. Black arrow indicates scanning direction, that is, from bone toward resin. Note that the P map appears “spotty” because the L‐edge of P was mostly within the tail of the plasmon peak, and limited signal could be extracted. Scale bars are 200 nm.

It is worth pointing out that the HAADF‐STEM images show that only a small region, overall less than 1 μm in width, at the bone–resin interface presented significant structural alterations. SEM–EDX and micro‐Raman spectroscopy may be unable to precisely characterize this region due to its limited width, especially in relation to the volume probed by these two techniques (183–257 μm^3^ in SEM–EDX and 0.7 μm^3^ in micro‐Raman spectroscopy), as well as to limitations in lateral and vertical resolution. However, variations in the mineral‐to‐matrix ratio suggest that compositional changes can occur over a broader area, so compositional analyses by SEM–EDX and micro‐Raman spectroscopy could effectively be capturing abnormalities due to the lack of ongoing mineralization and remodeling in MRONJ. Spectroscopy analyses with nanoscale resolution using EELS confirmed the decrease in Ca and P across the necrotic bone–resin interface (Figure 6), as was already evident from the variation in *Z*‐contrast in the HAADF‐STEM images (Figure 4).

### Overall considerations on the bone–bacteria interface in MRONJ

In light of the findings described in the preceding sections, we propose two mechanisms, likely concurrent, responsible for the creation of the hypermineralized and disordered regions at the interface between bone and the bacteria‐invaded region in MRONJ: (i) progressive mineralization, where a progressive, excessive mineralization occurs in the absence of cell‐mediated regulatory mechanisms; (ii) dissolution and reprecipitation, where bone mineral is, at least partly, degraded and dissolved by the bacteria and subsequently reprecipitates.

Evidence for the first mechanism stems from the fact that, given the necrosis, most cells in the tissue are dead, as seen from histology and resin cast etching. Bone cells play a fundamental regulatory role in coupling bone formation and resorption and in initiating/preventing mineralization.^(^ ^37^ ^)^ Therefore, the absence of viable cells can lead to the uncontrolled (both in terms of quantity and structural organization) precipitation of Ca and P at the bone–bacteria interface, especially since many body fluids, such as blood and saliva, are supersaturated with respect to calcium phosphate precipitation.^(^ ^38^, ^39^ ^)^ That mineralization is thermodynamically and kinematically favored in the absence of specific inhibitors is observed in pathological mineralization contexts such as atherosclerosis,^(^ ^40^ ^)^ kidney stones,^(^ ^41^ ^)^ and dental calculus.^(^ ^42^ ^)^ However, in an apparent contradiction, uncontrolled mineralization in MRONJ should still be prevented by the action of BPs because they are analogs of pyrophosphate (PPi), which are known mineralization inhibitors.^(^ ^43^ ^)^ On the other hand, an increased mineral content has been associated with MRONJ.^(^ ^4^, ^37^ ^)^

Regarding the second mechanism, although direct degradation of bone by biofilm pathogens is not yet widely recognized, the presence of bacteria at the interface with bone and of mineral‐like nanosized particulate matter within and around them (Figure 4E) supports an active role of bacteria in degrading bone, especially since osteoclast‐mediated resorption should be largely suppressed by BPs. The possibility of biofilm‐operated bone destruction has been proved in vitro and ex vivo,^(^ ^18^ ^)^ as well as post mortem in microbial bioerosion.^(^ ^44^, ^45^ ^)^ Gram‐negative bacteria, like those identified by microbial swab in this study, can adhere to proteins of the extracellular matrix (ECM), including collagen type I, facilitating further bacterial colonization and host invasion.^(^ ^46^ ^)^ Moreover, bacterial proteases can degrade ECM proteins, with bacterial collagenases specifically targeting collagen.^(^ ^47^ ^)^ Some of the disordered mineral at the interface could also correspond to Ca and P being released in the bone degradation process and reprecipitating. Moreover, Ca and P could also precipitate from the surrounding environment via the first mechanism described, enriching the region at the interface that appears as hypermineralized. BPs are known to inhibit the amorphous‐to‐crystalline transition of Ca and P precipitates,^(^ ^48^, ^49^ ^)^ which could explain the region lacking crystallinity identified by SAED (Figure 4D). Lastly, it cannot be excluded that, together with bacteria, host immune cells secondary to the presence of the pathogens also participate in the degradation of bone ECM. In fact, some multinucleated cells were observed in histological sections of necrotic bone, indicating the presence of inflammation (Figure S8). Additionally, since the administration of alendronate was interrupted 1.5 years before the sampling procedure, the presence of osteoclast‐mediated resorption cannot be excluded. However, as alendronate tablets were taken for a very extended period of time (23 years), it is reasonable to assume that osteoclastic activity is still largely suppressed, and some eventual osteoclastic resorption alone could not explain the changes we observed at the bone–bacteria interface. In addition, the absence of a mineralization front excludes the possibility that remodeling is taking place.

## Conclusions

As many have previously observed, MRONJ is a highly complex scenario involving multiple actors that may have direct or indirect effects, or both, on its onset and progression. Our structural and compositional analyses, focused on probing the bone–bacteria interface across multiple length scales, point to two main directions, involving nonmediated mineralization or a direct role of the bacteria. No matter the mechanism, it is evident that the necrotic bone is completely passive (after all, it is *dead*!), and the interface with the bacteria‐invaded space is a product of the environment outside it, whether that is the biofilm or a supersaturated Ca/P solution. Despite the systemic administration of BPs, a local effect emerges quite clearly, both macroscopically in the oral cavity, and at the micro‐ and nanoscale levels in bone. Macroscopically, there are areas in the jaw with no signs of necrosis or oral infection. When considering necrotic areas, the effect of MRONJ on bone micro‐/nanostructure and composition appears to be localized in a very limited region, smaller than 1 μm in width, right at the interface with the bacteria‐invaded region, giving rise to a hypermineralized band (on the bone side) and a highly disordered and amorphous area (on the bacteria‐invaded side).

## Author Contributions

**Chiara Micheletti:** Conceptualization; formal analysis; investigation; methodology; visualization; writing – original draft. **Liza‐Anastasia DiCecco:** Investigation; methodology; writing – review and editing. **Cecilia Larsson Wexell:** Conceptualization; resources; writing – review and editing. **Dakota M. Binkley:** Investigation; methodology; writing – review and editing. **Anders Palmquist:** Funding acquisition; supervision; writing – review and editing. **Kathryn Grandfield:** Funding acquisition; supervision; writing – review and editing. **Furqan Shah:** Conceptualization; funding acquisition; investigation; methodology; supervision; writing – review and editing.

## Authors' roles

CM: conceptualization, formal analysis, investigation, methodology, visualization, writing—original draft. L‐AD: investigation, methodology, writing—review and editing. CLW: conceptualization, resources, writing—review and editing. DMB: investigation, methodology, writing—review and editing. AP: funding acquisition, supervision, writing—review and editing. KG: funding acquisition, supervision, writing—review and editing. FAS: conceptualization, methodology, investigation, funding acquisition, supervision, writing—review and editing.

## Conflict of Interest

We declare that we have no conflict of interest.

### Peer Review

The peer review history for this article is available at https://publons.com/publon/10.1002/jbm4.10693.

## Supporting information


**Table S1** Values of width and slope of the bone–resin interface for C, Ca, and P measured from SEM‐EDX spectra.
**Table S2.** Values of mineral‐to‐matrix ratio measured for each of the points in the bone region in the micro‐Raman spectroscopy line scan.
**Figure S1.** (A, B) Panoramic X‐ray radiographies before (A) and after (B) tooth extraction surgery. (C, D) X‐ray radiographies corresponding to the frontal (C) and lateral (D) view of the upper jaw and maxillary sinus. (E) Picture of the surgery and bone sampling procedure, where areas of necrotic bone can be noted in the region marked by the white rectangle.
**Figure S2.** Histological images of necrotic (A–I) and non‐necrotic (J–L) bone. Images A, B, D, and G correspond to necrotic bone in the lower jaw; images C, E, F, H, and I correspond to necrotic bone in the upper jaw. Scale bars are 500 μm in A, 100 μm in B, C, D, E, F, J, and K, and 50 μm in G, H, I, and L. The following objectives were used: ×4 for image A; ×10 for images B, C, and J; ×20 for images D, E, F, and K; and ×40 for images G, H, I, and L.
**Figure S3.** Pseudo‐colored BSE‐SEM images of necrotic (A, B) and non‐necrotic (C) bone, corresponding to Figure 2G–I. A 16‐level lookup table has been applied after median filtering (“ndimage” module using a median filter with size 10 in “scipy” library in Python 3.8.10). Scale bars are 100 μm.
**Figure S4.** Osteocyte lacuna presenting signs of micropetrosis (BSE‐SEM image). Rhomboidal mineral nodules (arrowheads) likely correspond to magnesium whitlockite. Scale bar is 5 μm.
**Figure S5.** Additional SE‐SEM images of necrotic (A–I) and non‐necrotic (J–L) bone after resin cast etching. Images A, B, E, G, and H correspond to necrotic bone in the lower jaw; images C, D, F, and I correspond to necrotic bone in the upper jaw. Scale bars are 10 μm in A, B, C, and J, 5 μm in D, E, F, and K, and 2 μm in G, H, I, and L.
**Figure S6.** SAED pattern of the region where bone displays a normal ultrastructure (A), corresponding to the area marked by the green circle in the BF‐TEM image (B). The characteristic (002) arcs of *c*‐axis of hydroxyapatite are visible, as typically observed where collagen fibrils are in‐plane. Scale bar is 200 nm.
**Figure S7.** Unmarked HAADF‐STEM image corresponding to Figure 4E, showing bacteria in the resin region interfacing with necrotic bone. Three bacteria are indicated by arrowheads. Scale bar is 500 nm.
**Figure S8.** Histological section of necrotic bone where some multinucleated cells can be distinguished. Scale bar is 50 μm.

## Data Availability

The data used in this work are provided either in the main manuscript or in the supplementary information.
