# Supplementary material for: Multimodal and Multiscale Characterization of the Bone‐Bacteria Interface in a Case of Medication‐Related Osteonecrosis of the Jaw
Source: JBMR Plus. 2022 Nov 8;6(12):e10693. doi: 10.1002/jbm4.10693 (PMC9751653; doi:10.1002/jbm4.10693)
Supplement: Supplementary file 1 — Table S1 Values of width and slope of the bone–resin interface for C, Ca, and P measured from SEM‐EDX spectra. Table S2. Values of mineral‐to‐matrix ratio measured for each of the points in the bone region in the micro‐Raman spectroscopy line scan. Figure S1. (A, B) Panoramic X‐ray radiographies before (A) and after (B) tooth extraction surgery. (C, D) X‐ray radiographies corresponding to the frontal (C) and lateral (D) view of the upper jaw and maxillary sinus. (E) Picture of the surgery and bone sampling procedure, where areas of necrotic bone can be noted in the region marked by the white rectangle. Figure S2. Histological images of necrotic (A–I) and non‐necrotic (J–L) bone. Images A, B, D, and G correspond to necrotic bone in the lower jaw; images C, E, F, H, and I correspond to necrotic bone in the upper jaw. Scale bars are 500 μm in A, 100 μm in B, C, D, E, F, J, and K, and 50 μm in G, H, I, and L. The following objectives were used: ×4 for image A; ×10 for images B, C, and J; ×20 for images D, E, F, and K; and ×40 for images G, H, I, and L. Figure S3. Pseudo‐colored BSE‐SEM images of necrotic (A, B) and non‐necrotic (C) bone, corresponding to Figure 2G–I. A 16‐level lookup table has been applied after median filtering (“ndimage” module using a median filter with size 10 in “scipy” library in Python 3.8.10). Scale bars are 100 μm. Figure S4. Osteocyte lacuna presenting signs of micropetrosis (BSE‐SEM image). Rhomboidal mineral nodules (arrowheads) likely correspond to magnesium whitlockite. Scale bar is 5 μm. Figure S5. Additional SE‐SEM images of necrotic (A–I) and non‐necrotic (J–L) bone after resin cast etching. Images A, B, E, G, and H correspond to necrotic bone in the lower jaw; images C, D, F, and I correspond to necrotic bone in the upper jaw. Scale bars are 10 μm in A, B, C, and J, 5 μm in D, E, F, and K, and 2 μm in G, H, I, and L. Figure S6. SAED pattern of the region where bone displays a normal ultrastructure (A), corresponding to the area marked [file JBM4-6-e10693-s001.pdf]

SUPPLEMENTARY INFORMATION

Multimodal and Multiscale Characterization of the Bone-Bacteria Interface in a Case of Medication-Related Osteonecrosis of the Jaw

Chiara Micheletti[1,2], Liza-Anastasia DiCecco[1], Cecilia Larsson Wexell[2,3,4], Dakota M. Binkley[5], Anders Palmquist[2], Kathryn Grandfield[1,5], Furqan A. Shah[1,2*]

[1] Department of Materials Science and Engineering, McMaster University, Hamilton, ON, Canada

[2] Department of Biomaterials, Sahlgrenska Academy, University of Gothenburg, Gothenburg, Sweden

[3] Department of Oral and Maxillofacial Surgery, Skåne University Hospital, Lund, Sweden

[4] Department of Oral and Maxillofacial Surgery and Oral Medicine, Malmö University, Malmö, Sweden

[5] School of Biomedical Engineering, McMaster University, Hamilton, ON, Canada

* *Corresponding author:*

Furqan A. Shah

https://orcid.org/0000-0002-9876-0467

Email: furqan.ali.shah@biomaterials.gu.se

Tel: +46 31 786 28 98

Table S1. Values of width and slope of the bone-resin interface for C, Ca, and P measured from SEM-EDX spectra.

		Width C [μm]	Width Ca [μm]	Width P [μm]	Slope C [μm^{-1}]	Slope Ca [μm^{-1}]	Slope P [μm^{-1}]
**Necrotic sample (lower jaw)**	**line 1**	9.876	9.876	9.876	121.278	181.294	127.984
	**line 2**	12.546	13.741	14.338	130.320	113.090	80.197
	**line 3**	9.624	10.190	10.190	177.953	157.225	123.629
**Necrotic sample (upper jaw)**	**line 1**	9.340	11.416	10.897	142.552	138.464	114.936
	**line 2**	14.811	11.109	10.491	134.106	166.357	109.572
	**line 3**	13.014	13.780	14.545	119.835	133.857	95.977
**Non-necrotic sample (upper jaw)**	**line 1**	11.911	18.528	15.219	129.955	106.682	90.814
	**line 2**	14.720	17.318	15.586	63.2193	106.985	79.811
	**line 3**	9.048	13.572	10.179	150.193	138.915	114.873

Table S2. Values of mineral-to-matrix ratio measured for each of the points in the bone region in the micro-Raman spectroscopy line scan.

		line 1	line 2	line 3	line 4	line 5
**Necrotic sample (lower jaw)**	**point 1**	5.431	5.485	4.690	8.679	6.044
	**point 2**	5.135	3.670	4.978	7.261	4.016
	**point 3**	4.947	3.192	5.357	7.784	3.976
	**point 4**	4.525	3.425	4.066	5.821	5.667
	**point 5**	2.789	1.625	5.736	5.319	1.930
**Necrotic sample (upper jaw)**	**point 1**	4.647	11.273	8.668	4.473	6.876
	**point 2**	4.463	7.569	8.373	6.727	5.053
	**point 3**	5.388	7.510	11.919	4.638	11.284
	**point 4**	5.642	10.427	8.531	5.714	6.601
	**point 5**	6.357	7.330	11.771	5.969	6.361
**Non-necrotic sample (upper jaw)**	**point 1**	3.704	3.195	2.208	1.679	3.096
	**point 2**	3.334	2.678	1.939	1.744	3.056
	**point 3**	3.082	2.538	1.777	1.488	2.696
	**point 4**	2.322	2.202	1.583	1.430	2.740
	**point 5**	1.299	1.724	1.484	1.192	2.800

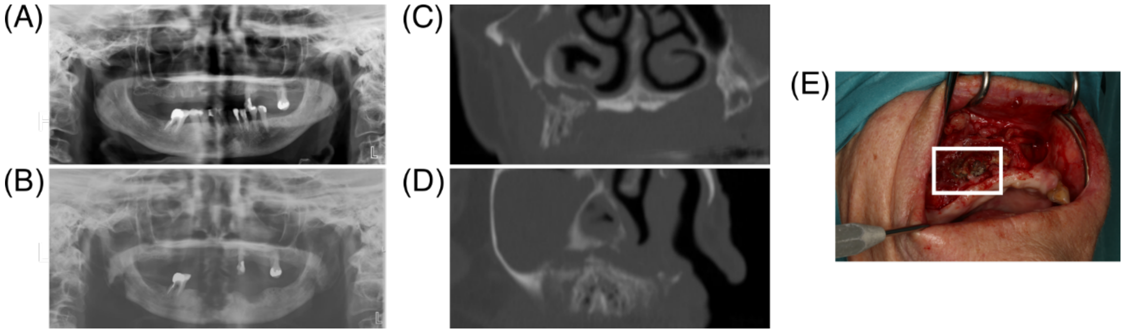

Figure S1. (A, B) Panoramic X-ray radiographies before (A) and after (B) tooth extraction surgery. (C, D) X-ray radiographies corresponding to the frontal (C) and lateral (D) view of the upper jaw and maxillary sinus. (E) Picture of the surgery and bone sampling procedure, where areas of necrotic bone can be noted in the region marked by the white rectangle.

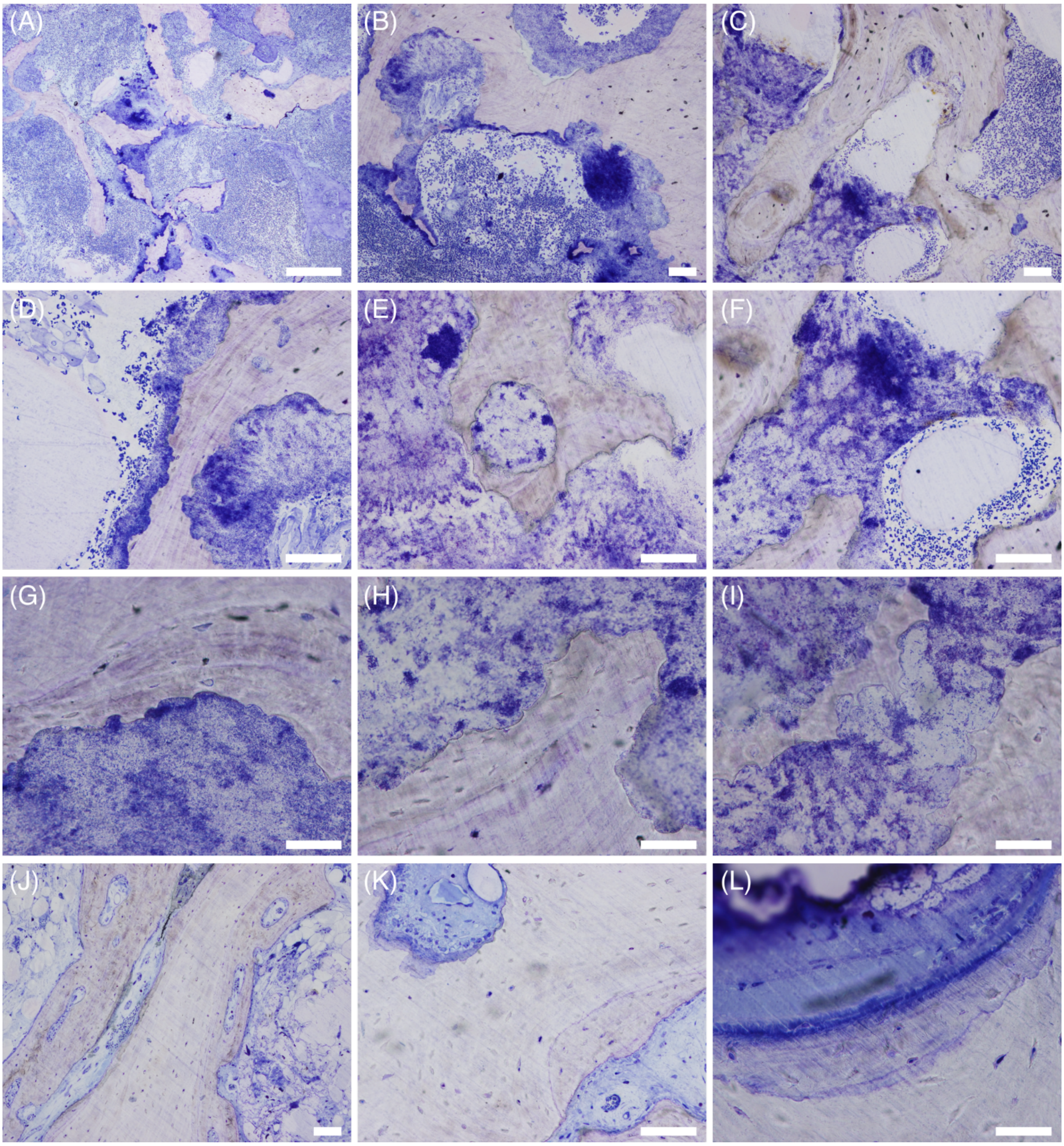

Figure S2. Histological images of necrotic (A–I) and non-necrotic (J–L) bone. Images A, B, D, and G correspond to necrotic bone in the lower jaw; images C, E, F, H, and I correspond to necrotic bone in the upper jaw. Scale bars are 500 µm in A, 100 µm in B, C, D, E, F, J, and K, and 50 µm in G, H, I, and L. The following objectives were used: ×4 for image A; ×10 for images B, C, and J; ×20 for images D, E, F, and K; and ×40 for images G, H, I, and L.

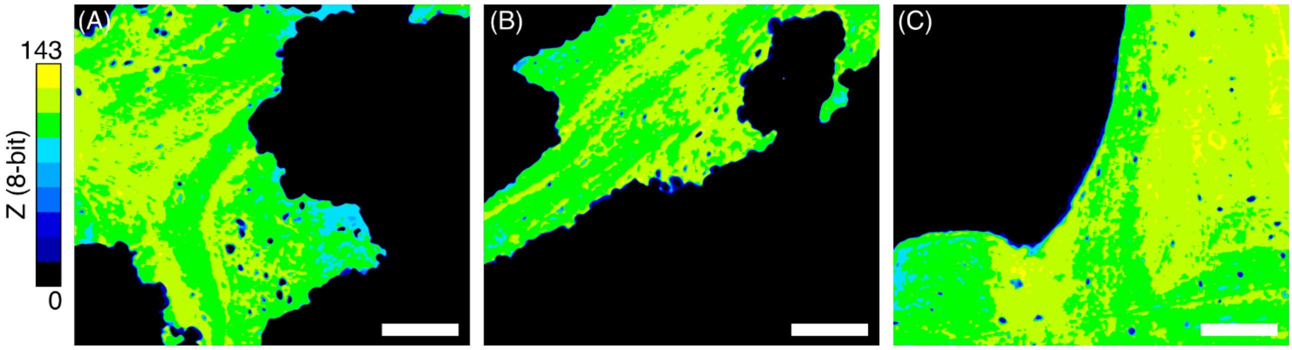

Figure S3. Pseudo-coloured BSE-SEM images of necrotic (A, B) and non-necrotic (C) bone, corresponding to Figure 2G–I. A 16-level lookup table has been applied after median filtering (“ndimage” module using a median filter with size 10 in the “scipy” library in Python 3.8.10). Scale bars are 100 µm.

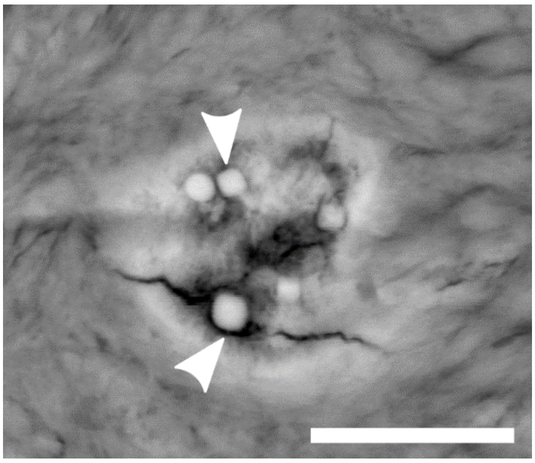

Figure S4. Osteocyte lacuna presenting signs of micropetrosis (BSE-SEM image). Rhomboidal mineral nodules (arrowheads) likely correspond to magnesium whitlockite. Scale bar is 5 µm.

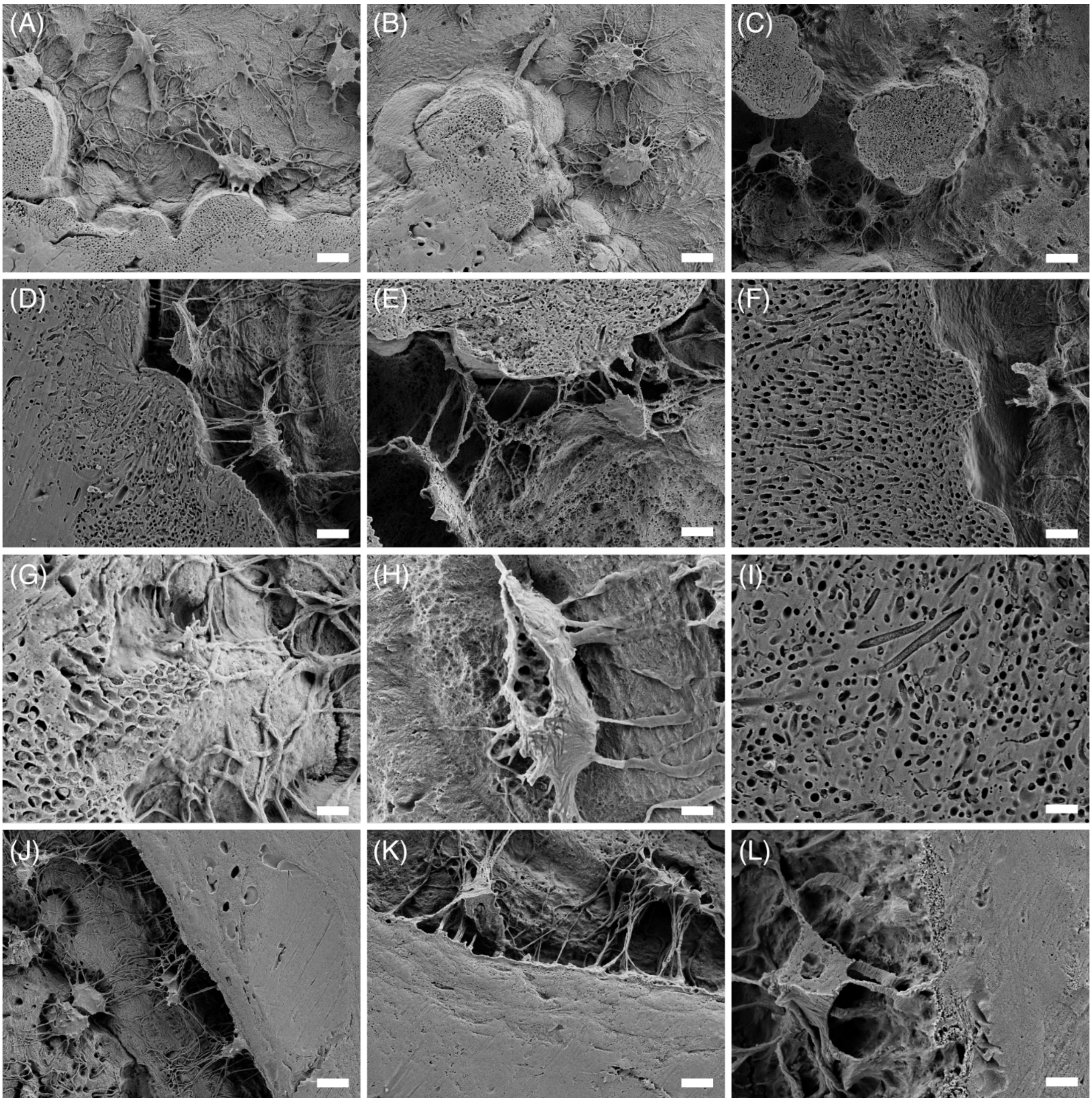

Figure S5. Additional SE-SEM images of necrotic (A–I) and non-necrotic (J–L) bone after resin cast etching. Images A, B, E, G, and H correspond to necrotic bone in the lower jaw; images C, D, F, and I correspond to necrotic bone in the upper jaw. Scale bars are 10 μm in A, B, C, and J, 5 μm in D, E, F, and K, and 2 μm in G, H, I, and L.

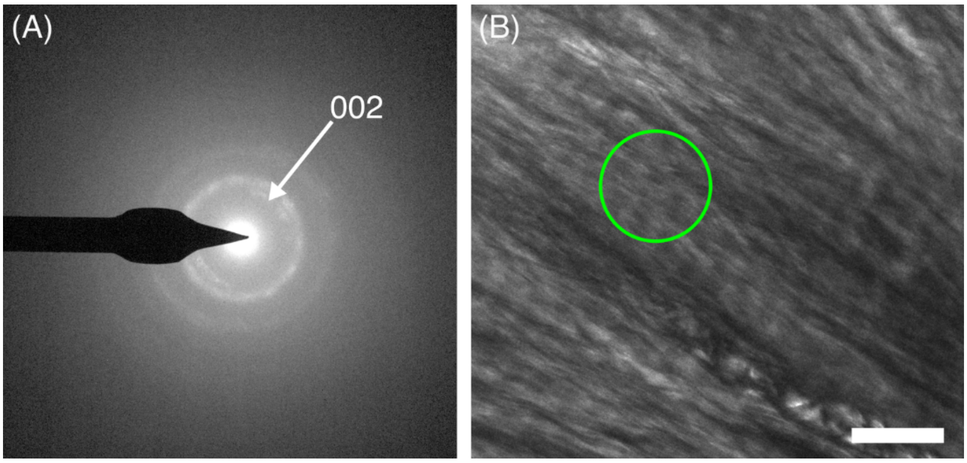

Figure S6. SAED pattern of the region where bone displays a normal ultrastructure (A), corresponding to the area marked by the green circle in the BF-TEM image (B). The characteristic (002) arcs of the *c*-axis of hydroxyapatite are visible, as typically observed where collagen fibrils are in-plane. Scale bar is 200 nm.

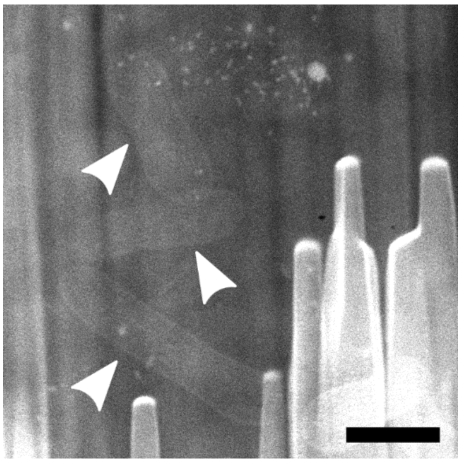

Figure S7. Unmarked HAADF-STEM image corresponding to Figure 4E, showing bacteria in the resin region interfacing with necrotic bone. Three bacteria are indicated by arrowheads. Scale bar is 500 nm.

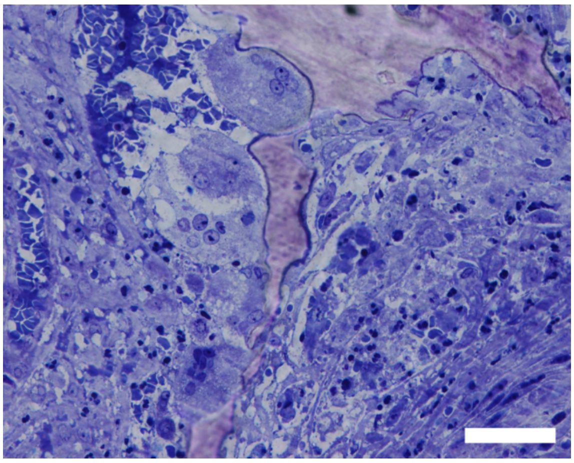

Figure S8. Histological section of necrotic bone where some multinucleated cells can be distinguished. Scale bar is 50 µm.
